# Supplementary material for: SIRT1-Mediated Epigenetic Protective Mechanisms of Phytosome-Encapsulated Zea mays L. var. ceratina Tassel Extract in a Rat Model of PM2.5-Induced Cardiovascular Inflammation
Source: Int J Mol Sci. 2025 Jun 16;26(12):5759. doi: 10.3390/ijms26125759 (PMC12193228; doi:10.3390/ijms26125759)
Supplement: Supplementary file 1 [file ijms-26-05759-s001.zip › ijms-3668623-supplementary.pdf]

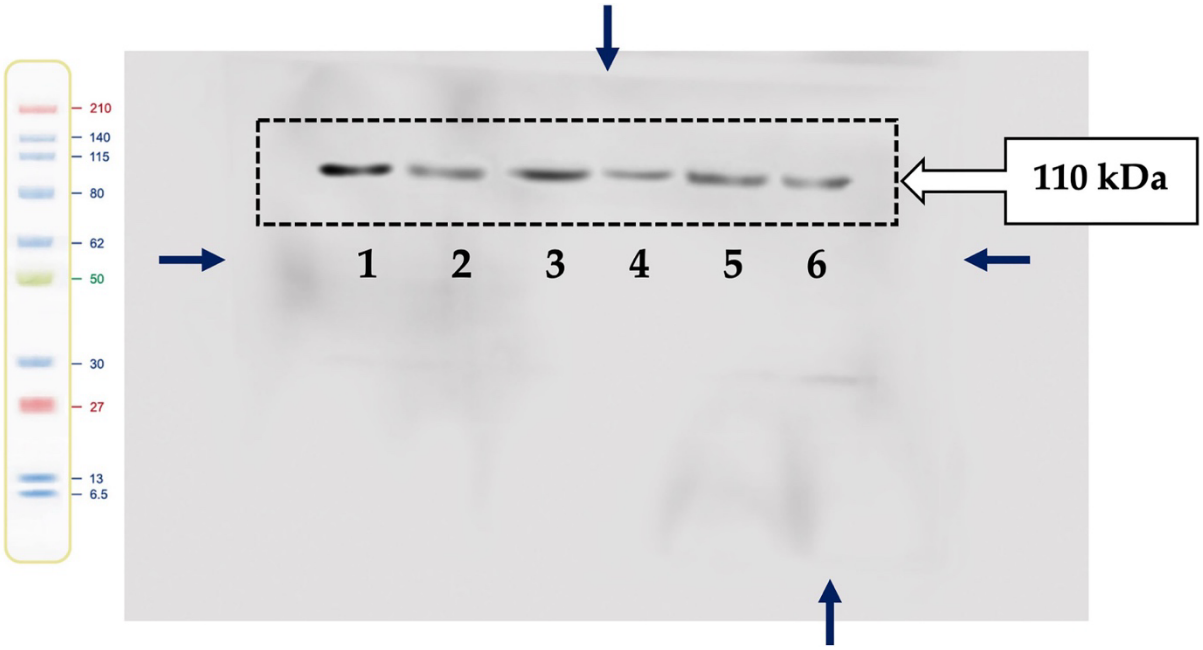

Figure S1: Western blotting was used to detect SIRT1 expression (110 kDa) in cardiac tissue. The groups included: (1) Naïve control, (2) PM2.5 exposure + vehicle, (3) PM2.5 exposure + prednisolone (1 mg/kg BW), (4) PM2.5 exposure + PZT (100 mg/kg BW), (5) PM2.5 exposure + PZT (200 mg/kg BW), and (6) PM2.5 exposure + PZT (400 mg/kg BW). The full-length membranes are shown, with membrane edges indicated by dark blue arrows.

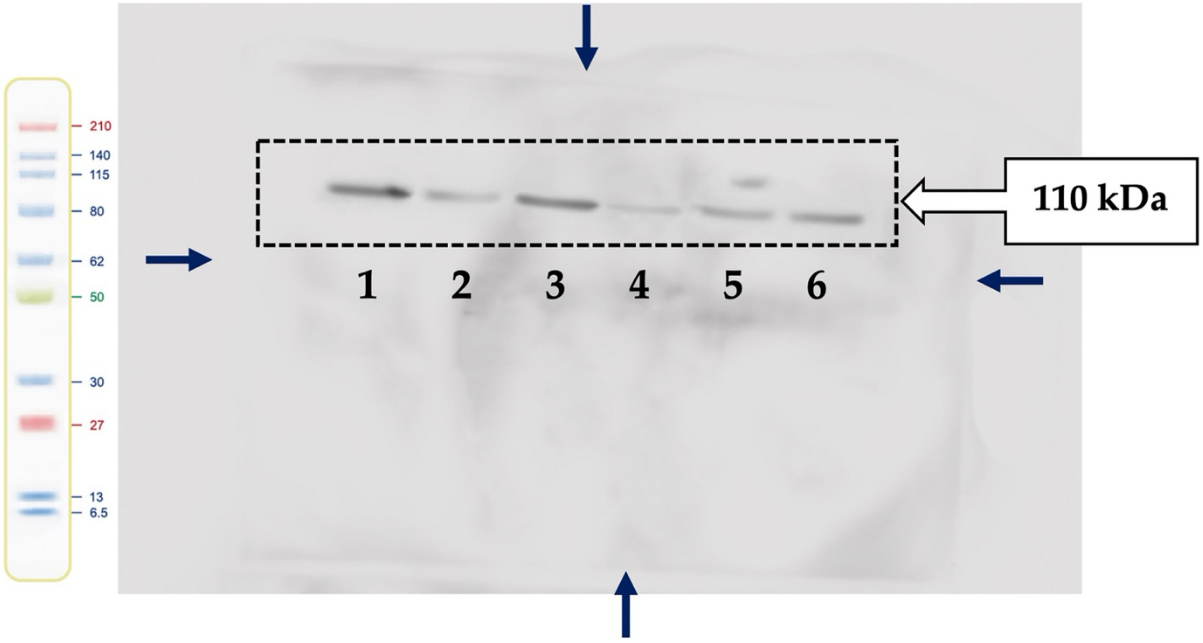

Figure S2: Western blotting was used to detect SIRT1 expression (110 kDa) in vascular tissue. The groups included: (1) Naïve control, (2) PM2.5 exposure + vehicle, (3) PM2.5 exposure + prednisolone (1 mg/kg BW), (4) PM2.5 exposure + PZT (100 mg/kg BW), (5) PM2.5 exposure + PZT (200 mg/kg BW), and (6) PM2.5 exposure + PZT (400 mg/kg BW). The full-length membranes are shown, with membrane edges indicated by dark blue arrows.

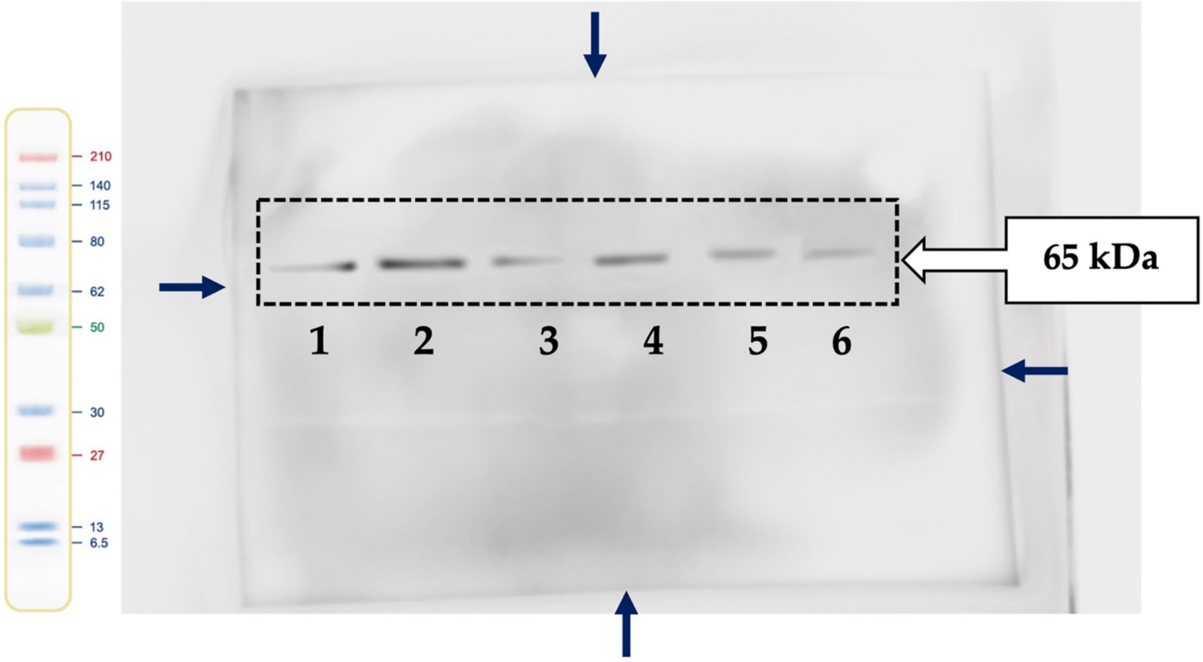

Figure S3: Western blotting was used to detect NF-κB expression (65 kDa) in cardiac tissue. The groups included: (1) Naïve control, (2) PM2.5 exposure + vehicle, (3) PM2.5 exposure + prednisolone (1 mg/kg BW), (4) PM2.5 exposure + PZT (100 mg/kg BW), (5) PM2.5 exposure + PZT (200 mg/kg BW), and (6) PM2.5 exposure + PZT (400 mg/kg BW). The full-length membranes are shown, with membrane edges indicated by dark blue arrows.

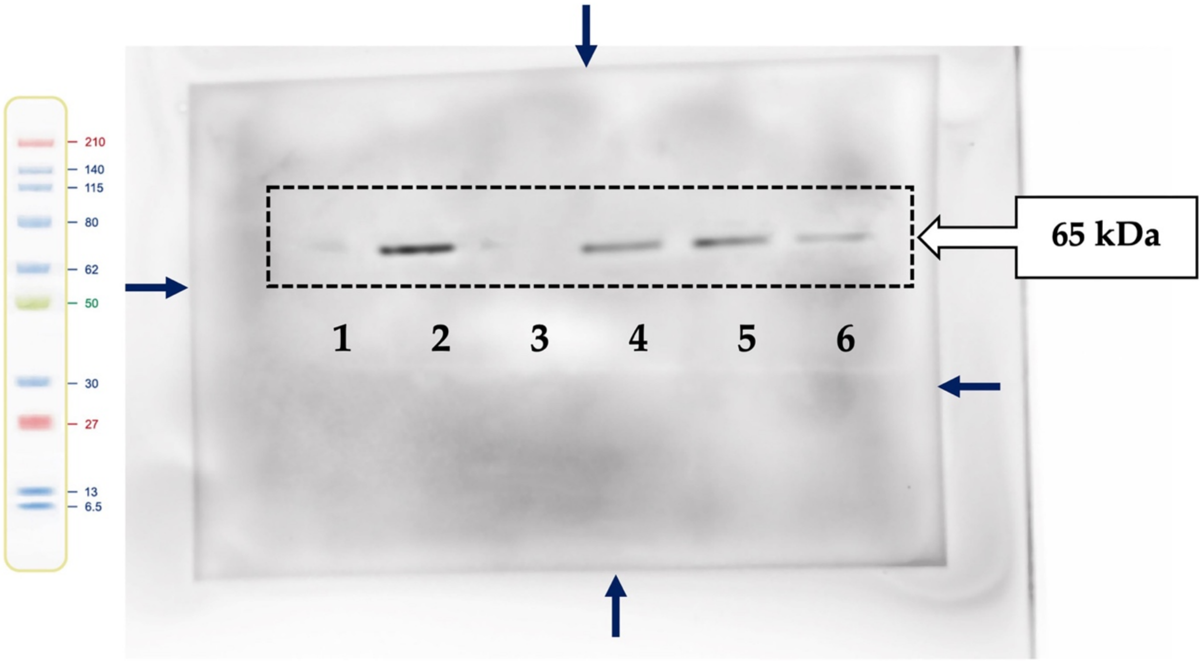

Figure S4: Western blotting was used to detect NF-κB expression (65 kDa) in vascular tissue. The groups included: (1) Naïve control, (2) PM2.5 exposure + vehicle, (3) PM2.5 exposure + prednisolone (1 mg/kg BW), (4) PM2.5 exposure + PZT (100 mg/kg BW), (5) PM2.5 exposure + PZT (200 mg/kg BW), and (6) PM2.5 exposure + PZT (400 mg/kg BW). The full-length membranes are shown, with membrane edges indicated by dark blue arrows.

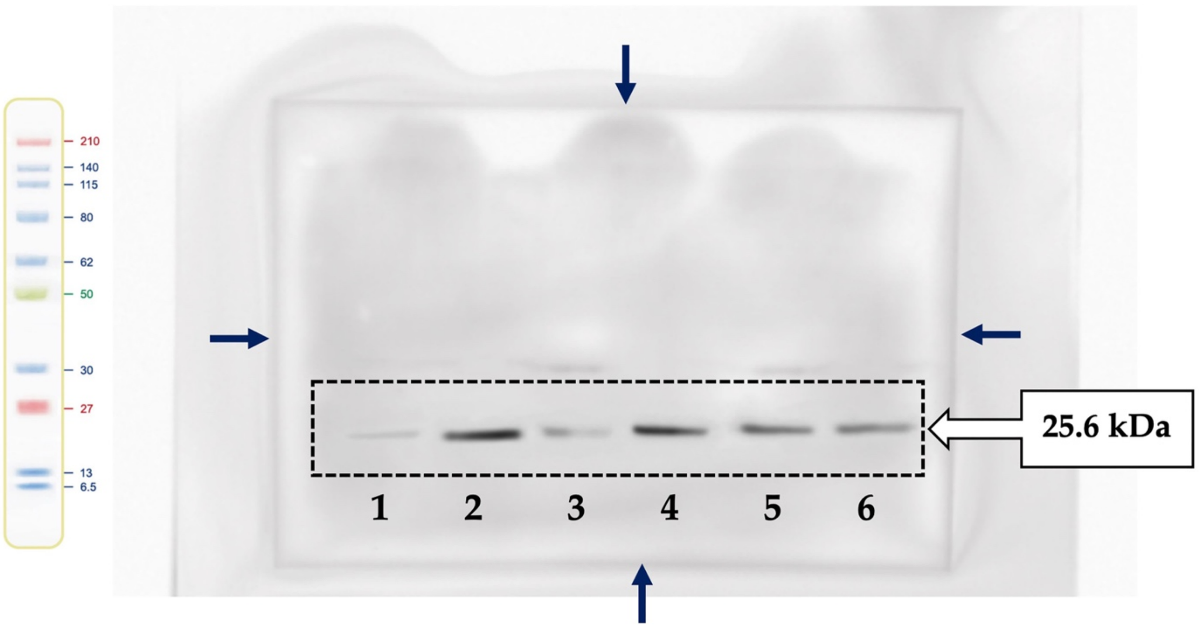

Figure S5: Western blotting was used to detect TNF-α expression (25.6 kDa) in cardiac tissue. The groups included: (1) Naïve control, (2) PM2.5 exposure + vehicle, (3) PM2.5 exposure + prednisolone (1 mg/kg BW), (4) PM2.5 exposure + PZT (100 mg/kg BW), (5) PM2.5 exposure + PZT (200 mg/kg BW), and (6) PM2.5 exposure + PZT (400 mg/kg BW). The full-length membranes are shown, with membrane edges indicated by dark blue arrows.

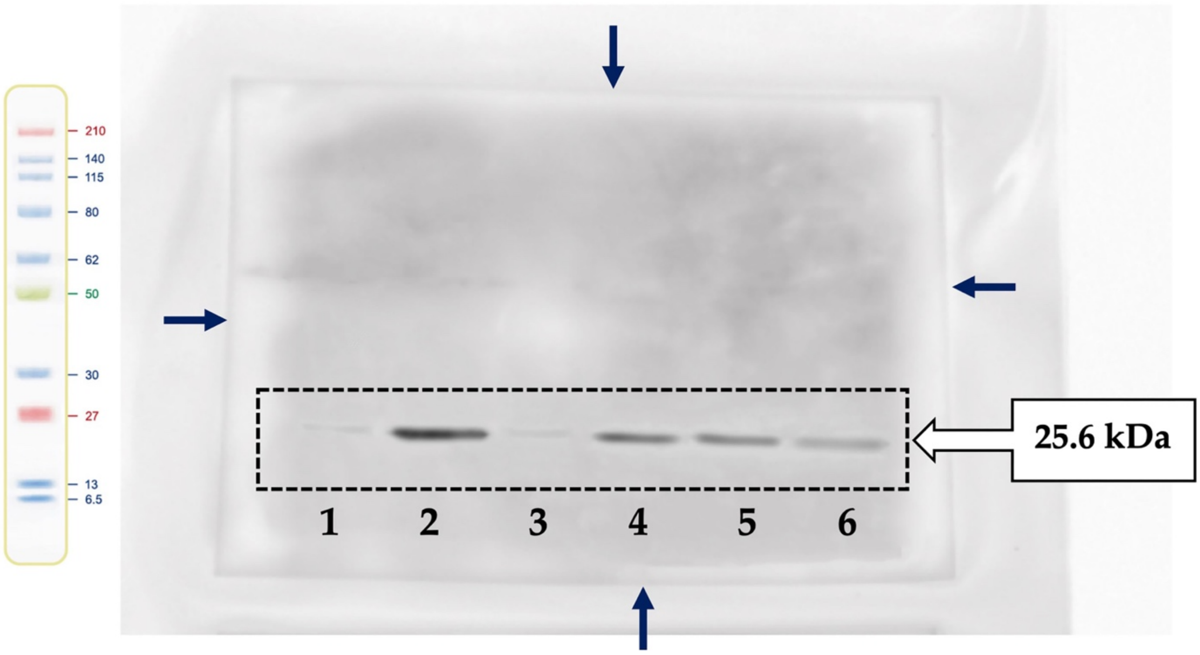

Figure S6: Western blotting was used to detect TNF-α expression (25.6 kDa) in vascular tissue. The groups included: (1) Naïve control, (2) PM2.5 exposure + vehicle, (3) PM2.5 exposure + prednisolone (1 mg/kg BW), (4) PM2.5 exposure + PZT (100 mg/kg BW), (5) PM2.5 exposure + PZT (200 mg/kg BW), and (6) PM2.5 exposure + PZT (400 mg/kg BW). The full-length membranes are shown, with membrane edges indicated by dark blue arrows.

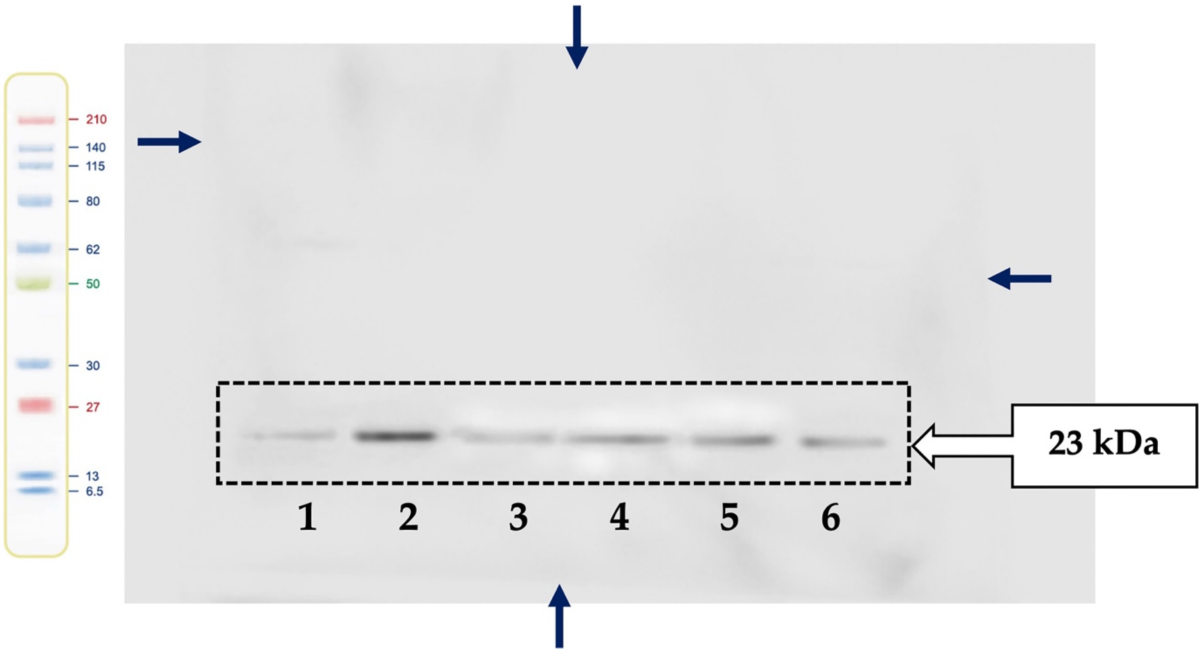

Figure S7: Western blotting was used to detect IL-6 expression (23 kDa) in cardiac tissue. The groups included: (1) Naïve control, (2) PM2.5 exposure + vehicle, (3) PM2.5 exposure + prednisolone (1 mg/kg BW), (4) PM2.5 exposure + PZT (100 mg/kg BW), (5) PM2.5 exposure + PZT (200 mg/kg BW), and (6) PM2.5 exposure + PZT (400 mg/kg BW). The full-length membranes are shown, with membrane edges indicated by dark blue arrows.

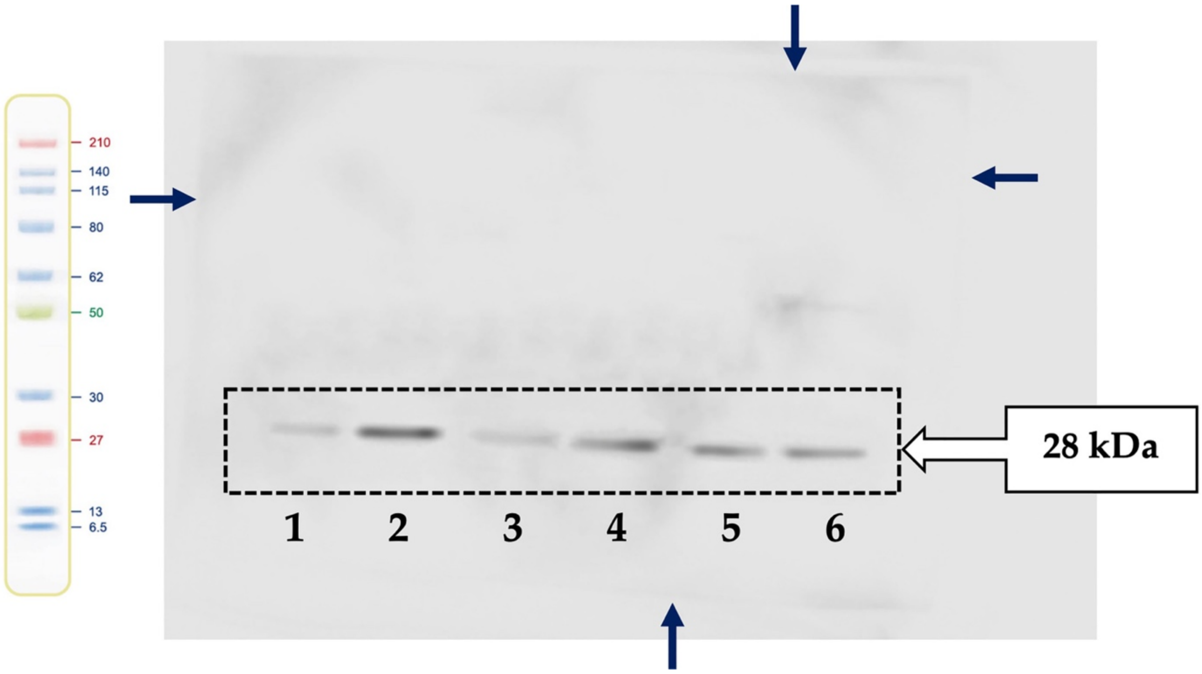

Figure S8: Western blotting was used to detect TGF-β expression (28 kDa) in vascular tissue. The groups included: (1) Naïve control, (2) PM2.5 exposure + vehicle, (3) PM2.5 exposure + prednisolone (1 mg/kg BW), (4) PM2.5 exposure + PZT (100 mg/kg BW), (5) PM2.5 exposure + PZT (200 mg/kg BW), and (6) PM2.5 exposure + PZT (400 mg/kg BW). The full-length membranes are shown, with membrane edges indicated by dark blue arrows.
